# Supplementary material for: Chromothripsis during telomere crisis is independent of NHEJ, and consistent with a replicative origin
Source: Genome Res. 2019 May;29(5):737–49. doi: 10.1101/gr.240705.118 (PMC6499312; doi:10.1101/gr.240705.118)
Supplement: Supplemental Material [file supp_gr.240705.118_Supplemental_file_1.zip › contigs/annotated_contigs/DB56/contig.4.DB56_length_752_mean_cov_11.9348404255.docx]

**DB56_length_752_mean_cov_11.9348404255**

```
CCCC|CTCCCCCTCCCCCTCCCCCTGCCCCCCA|CCCCCCT|CTTTTTTTTTTTTTTTTTTTGAGACGGAGTCTTGCTCTGTCACCCAG
     >chr19:23601421-23601449 + E=5e-02 p=4e-02  >chr16:2481622-2481700 - E=4e-23 p=3e
GCTGGAGTGCAATGCTGTGATCTCGGCTCAC|CGCCCCACCTGCGCCTCCTGA|GTTCAAGTGATTCTTCTGCCTCAGCCTCCTGAGTA
-02                                                  >chr18:55087827-55088149 + E=8e-14
ACTGGGATTACAGGCGCCCACCACCACGGCTGGCTAATTTTTGTATTTTTAGTAGAGATGGGGTTTCACCATGTTGGTCAGGCTGGTCT
9
TGAACTCCCGACCTCAGGTGATCCACCCACCTCGACCTCCCAAAGTGCTGGGATTACAGGCATAAGCCACCACGCCCAGCCCAGGTAGT

TCTTTATAGAAGTGTGAGAATGGACTAATACACTACCCAAAATGATCTACACATGGAATGCAATCCCTAGAAAAATACCAATGACATTC

TTCACAGAAGTAGAAAAAA|C|TAAAATATTTATCTAAGGGGTCTGATTTATCAACCTCAGGTAGTCTCCAAAACACAAAGGTATTCAG
                    >chr18:55032844-55033137 + E=2e-164
GCTAAAATAAAACCAATCAAAAAAGGAAATTCAGGAGGCTCCAGTTCTAAGACTAACCCAGTATAACAAGGGTGATCAGTTATTACTTG

TGGCCTTGTACCACCTCTGTTGGATAATGTGCCACCGTCCTAATGAAGCTTTGGCCAGACTTCTTTCTATAGCTGTGCACATAGAAAGG

CTTTAGACAACAGTGATACCAAAGGGTTCCTTTGATTTTCCTTCTGT
```
